# Genomic signatures in *Variovorax* enabling colonization of the *Populus* endosphere

**DOI:** 10.1128/msystems.01605-25

**Published:** 2026-01-16

**Authors:** Delaney G. Beals, Dana L. Carper, Leah H. Hochanadel, Sara S. Jawdy, Dawn M. Klingeman, Bryan T. Piatkowski, David J. Weston, Mitchel J. Doktycz, Dale A. Pelletier

**Affiliations:** 1 Biosciences Division, Oak Ridge National Laboratory 6146, Oak Ridge, Tennessee, USA; 2 Department of Quantitative Health Sciences, Mayo Clinic 6915, Rochester, Minnesota, USA; Universiteit Leiden, Leiden, Netherlands

**Keywords:** endosphere colonization, strain-resolved metagenomics, comparative genomics, niche differentiation, synthetic community, functional trait enrichment

## Abstract

Microbial colonization of plant roots involves strong selective pressures that shape the structure and function of root-associated communities. In particular, the endosphere represents a highly selective environment requiring host entry and *in planta* persistence. However, strain-specific microbial traits that enable endosphere colonization remain poorly understood. Here, we use a defined, genome-resolved community of 28 *Variovorax* strains isolated from the roots of *Populus deltoides* and *Populus trichocarpa* (poplar trees) to determine which strains partition between rhizosphere and endosphere compartments and to identify the genomic traits associated with endosphere specialization. By combining strain-resolved metagenomic profiling, comparative genomics, and functional assays, we demonstrate that dominant endosphere colonizers are enriched in genes related to nutrient metabolism, redox balance, transcriptional regulation, and a conserved L-fucose utilization pathway experimentally shown to enhance root colonization. Not all strains succeed through the same strategy. Community-wide functional profiling revealed a distinct and reduced set of traits in the endosphere, including orthogroups associated with low-abundance strains that were overlooked in strain-level analyses. These findings reveal that multiple ecological strategies, such as metabolic competition, regulatory adaptation, and niche specialization, can support endosphere colonization. Our results advance the understanding of how bacterial colonization traits are distributed and deployed within a plant microbiome and suggest that host filtering selects for distinct, and sometimes complementary, microbial strategies. This work supports a shift toward mechanistic, genome-resolved models of microbiome assembly and offers a framework for linking microbial function to host colonization success.

**IMPORTANCE:**

Plants often depend on diverse microbial partners to support their growth, resilience, and adaptation to changing environments. Among these microbes, some bacteria inhabit the rhizosphere (the narrow zone around roots where microbes interact with the plant) while others are able to enter and persist within root tissues. The traits that distinguish these two lifestyles remain poorly understood. In this study, we examined a group of related *Variovorax* strains from poplar tree root microbiomes to ask why some rhizosphere-associated strains also become successful endosphere colonizers. We found that strains appear to succeed through different strategies: some may benefit from rapid growth on plant-derived carbon sources, while others may rely on stress tolerance or fine-tuned regulation. These results suggest that there is no single path from the rhizosphere into the root interior, but rather multiple strategies shaped by the host environment. Understanding this diversity can inform efforts to design resilient plant-microbe communities.

## INTRODUCTION

Plant-associated microbial communities play a central role in plant growth, health, and environmental responsiveness (1–4). Among these communities, root-associated bacteria inhabiting the rhizosphere and endosphere are particularly important. The rhizosphere, a carbon-rich zone surrounding the plant root, is influenced by root exudates and supports a diverse and dynamic assemblage of microbes, with individual plants often hosting between 50 and 1,000 bacterial operational taxonomic units (OTUs) (5, 6). A subset of these microbes can further colonize internal root tissues as endophytes, forming close associations with the host that may confer additional benefits to the plant (7).

Compared to the rhizosphere, the endosphere harbors lower microbial diversity, suggesting strong ecological filtering by the host. Colonization of the endosphere represents an ecological selection bottleneck, requiring bacteria not only to reach the root interior but also to survive the selective pressures of the host environment, establish themselves amid competing microbes, and maintain a functional presence long enough to influence plant physiology (8–11). Endophytes that pass through these ecological filters are often associated with enhanced host growth, increased abiotic stress tolerance, and protection against pathogens (12, 13). Despite the importance of these benefits, we still lack the framework for predicting which rhizosphere-associated bacteria can successfully colonize the endosphere, and what traits enable them to do so.

Prior studies have identified a wide range of bacterial traits that facilitate plant root colonization, including chemotaxis, secretion systems, and metabolic versatility (12, 14–16). More detailed studies in specific systems, such as tomato plant root tip colonization, have revealed that competitive colonization is shaped by a combination of metabolic capabilities, regulatory systems, cell surface structures, and secretion pathways (17, 18). While these findings highlight the multifactorial nature of bacterial adaptation to the root environment, most have been framed at a broad taxonomic scale, leaving open how specific genetic features influence niche preference within root-associated habitats. In particular, little is known about how these adaptations vary among closely related strains within the same genus or how that variation contributes to differential colonization of the rhizosphere versus the endosphere.

To address these gaps, we aimed to identify bacterial genomic signatures associated with endosphere colonization from the rhizosphere and test whether they can explain differences in ecological behavior among closely related strains. Developing such a framework is key for predicting microbiome assembly from genomic content and ultimately for rationally designing beneficial microbial consortia. We focused on *Variovorax*, a bacterial genus frequently identified as a core member of root microbiomes across diverse plant hosts, including *Populus* (19), *Arabidopsis* (20), and *Brassica* (21). Although *Variovorax* strains are often characterized as plant growth-promoting bacteria (22–26), their relative preference to colonize internal root tissues varies substantially, suggesting functional differentiation within the genus. This makes *Variovorax* a useful system for dissecting the genomic and ecological bases of niche specialization.

In this study, we examine *Variovorax* bacterial strains previously isolated from the rhizosphere, root surface, and endosphere of *Populus deltoides* and *Populus trichocarpa* trees growing in natural environments. Because these strains originate from natural *Populus* root environments, they represent ecologically relevant variation in host-associated traits. We therefore hypothesized that endosphere isolates may retain functions that facilitate root colonization under controlled experimental conditions. Building on this hypothesis, we aimed to determine how individual plant-associated *Variovorax* lineages differ in their colonization patterns across root compartments and what genomic traits distinguish endosphere-competent strains.

To test this, we assembled a defined community (DefCom) of 28 fully sequenced *Variovorax* strains (19) and inoculated axenic *Populus* plants, tracking colonization outcomes with strain-resolved metagenomics. This design parallels the “select-and-resequence” framework developed for plant-associated bacterial systems (27, 28), adapted here to quantify strain-resolved colonization dynamics within the *Populus* root microbiome. This community-based approach enabled high-throughput assessment of strain performance under direct competition in a shared host environment, providing a scalable alternative to pairwise inoculation assays. By integrating comparative genomics with orthogroup-level functional profiling, we identified both conserved and strain-specific determinants of endosphere colonization and experimentally validated one such function, L-fucose metabolism. These findings highlight the complexity of microbial adaptation to plant-associated niches and demonstrate how genome content can be linked to ecological outcomes at the strain level.

## RESULTS

### *Populus* hosts are colonized by diverse *Variovorax* lineages

**Fig 1:**
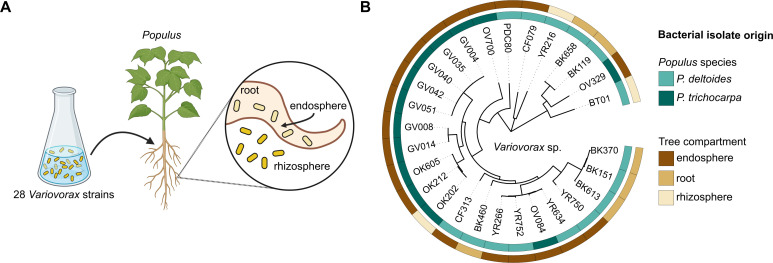
(**A**) Experimental overview of DefCom inoculation and root colonization. A defined community of 28 *Variovorax* strains isolated from *Populus* roots was introduced into sterile *Populus* microcosms. Colonization in the rhizosphere and endosphere was assessed after 3 weeks by strain-resolved metagenomic mapping. (**B**) Maximum-likelihood phylogeny of *Variovorax* isolates based on 1,147 single-copy orthologs. Colored boxes indicate the *Populus* genotype and plant compartment from which each strain was isolated (19).

To determine which members of the *Variovorax* DefCom preferentially colonize *Populus* roots, we inoculated axenic *P. deltoides* and *P. trichocarpa* plants with a defined 28-strain community ( Table S1) and tracked colonization in the rhizosphere and endosphere after 3 weeks of growth (Fig. 1A). The strains represent a broad range of phylogenetic diversity within the genus, spanning multiple clades of the *Variovorax* tree (Fig. 1B), including several closely related groups and more distantly related lineages based on average nucleotide identity (Fig. S1). Nineteen of the 28 strains were originally isolated from *Populus* endospheres in natural environments, while the remainder were recovered from roots and rhizospheres. These source designations provide contextual information but are not interpreted as fixed ecological categories, as an isolation source alone cannot resolve the genetic basis of endosphere adaptation. Because many *Variovorax* genomes in the DefCom share high nucleotide identity, metagenomic reads were mapped to all DefCom reference genomes using unique mapping to prevent double-counting of reads that align equally well to multiple strains.

### Endosphere colonization outcomes are dominated by a small subset of *Variovorax* strains

**Fig 2:**
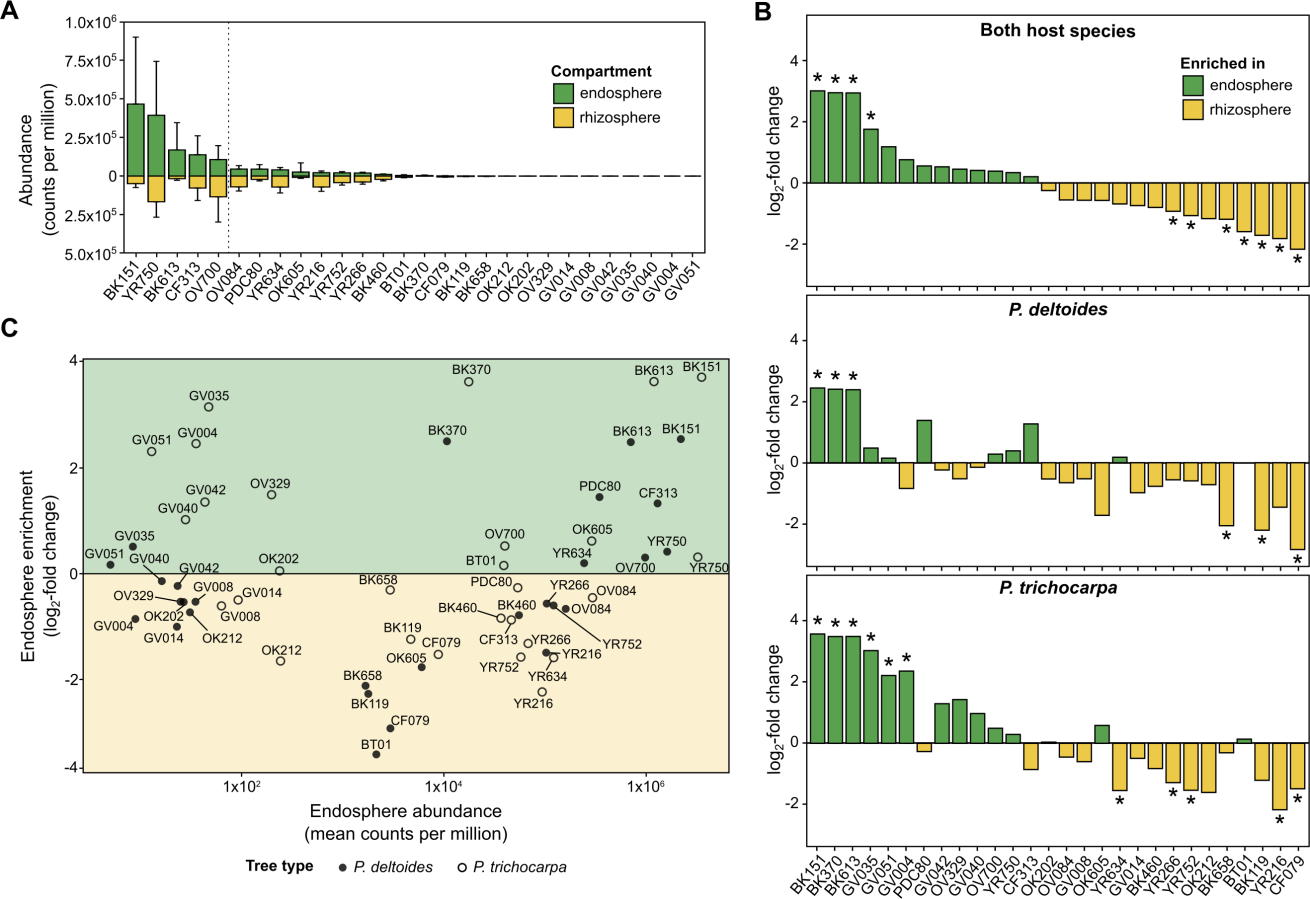
(**A**) Mean strain abundance (counts per million, CPM ± SD) in the endosphere (*n* = 7) and rhizosphere (*n* = 8) across *Populus* species. Strains are ordered by descending endosphere abundance; those to the left of the vertical dotted line together account for ~80% of all endosphere reads. (**B**) Differential abundance of strains in the endosphere versus rhizosphere of *P. deltoides* and *P. trichocarpa*. Bars show log₂ fold change (endosphere vs. rhizosphere) based on CPM-normalized data, representing relative enrichment between compartments; asterisks indicate significant enrichment after false discovery rate (FDR) correction (FDR < 0.05). Positive values denote endosphere enrichment, and negative values denote rhizosphere enrichment. (**C**) Relationship between strain abundance and compartment enrichment. Individual points represent strains; filled circles correspond to values from *P. deltoides* microcosms and open circles to *P. trichocarpa*.

Despite equal starting abundances, endosphere colonization outcomes were uneven: five strains (BK151, YR750, BK613, CF313, and OV700) accounted for ~80% of total mapped reads in the endosphere compartment (Fig. 2A). To examine how strains partition between root compartments, we calculated the log₂ fold change in normalized abundance between the endosphere and rhizosphere for each strain in both *P. deltoides* and *P. trichocarpa* (Fig. 2B), representing their relative enrichment between compartments. Across hosts, BK151, BK370, BK613, and GV051 were significantly enriched in the endosphere. In *P. deltoides*, enrichment was the strongest for BK151, BK370, and BK613, whereas in *P. trichocarpa,* additional GV lineage strains (GV035, GV051, and GV004) were significantly enriched. Several other strains exhibited host-specific enrichment patterns that paralleled their isolation source (Fig. 1B), with *P. deltoides* isolates (CF313, PDC80, and YR634) tending to be more abundant in *P. deltoides* roots, while GV lineage strains (GV035, GV051, and GV004) were more enriched in *P. trichocarpa*, although these differences were not significant.

### Host-specific enrichment patterns suggest multiple colonization strategies

To assess how abundance and enrichment together describe endosphere colonization behavior across *Populus* hosts, we compared strain-level abundance against log₂ fold change between the endosphere and rhizosphere (Fig. 2C). This analysis revealed three general colonization strategies. Strong colonizers such as BK151, BK370, and BK613 combined moderate-to-high endosphere abundance with strong endosphere enrichment in both tree species. Niche specialists, including OV329 in *P. trichocarpa*, were endosphere-enriched despite moderate abundance, while generalists such as YR750 and CF313 maintained high abundance in both endosphere and rhizosphere compartments.

Together, these patterns identified six strains (CF313, OV700, YR750, BK370, BK151, and BK613) as the dominant endosphere-associated members of the DefCom community. However, neither isolation history nor phylogenetic relatedness predicted their success. Only three of these strains were originally recovered from *Populus* endospheres, and pairwise average nucleotide identity (ANI) comparisons showed that strong colonizers were distributed both within a highly similar (≥98.5%) ANI cluster and across more divergent clades (Fig. S1). Although formal species boundaries have not been established for *Variovorax*, these values indicate that endosphere competence varies within and between putative species and reflects specific functional traits rather than taxonomic origin.

To resolve these traits, we carried out a comparative genomic analysis using ANI-defined lineages. We applied a ≥97% ANI threshold to group all 28 DefCom strains into lineages (Fig. S1), preventing near-identical genomes from being treated as independent observations. These lineage assignments established the evolutionary units used to identify genomic features associated with endosphere colonization.

### Comparative genomics reveals common traits among dominant endosphere colonizers

**Fig 3:**
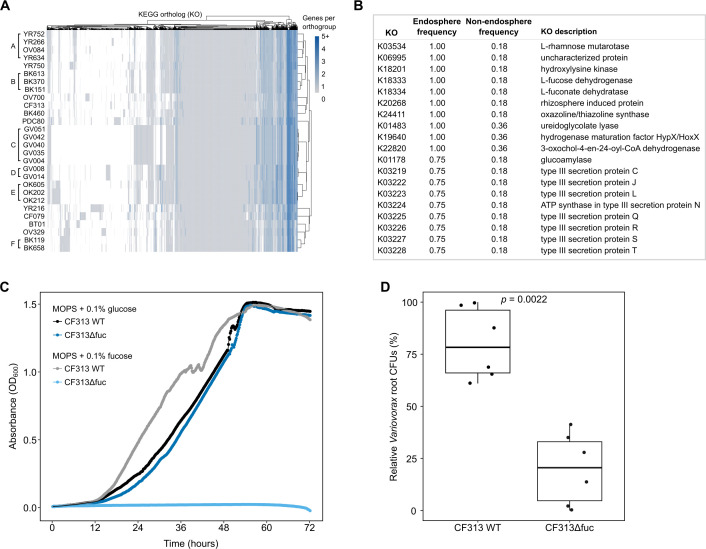
(**A**) Heatmap of the number of genes annotated to each KO across DefCom *Variovorax* strains. Columns show KOs detected in at least one genome; rows show strains used in this study. Color intensity indicates the number of unique genes annotated with each KO per strain (white = absent, dark blue = higher counts); capped at five genes for visualization. Letter labels on the left denote *Variovorax* shared lineages (≥97% ANI); unlabeled strains represent unique lineages. (**B**) Top 20 KOs enriched in the *Populus* endosphere based on lineage-level frequency. Endosphere-associated lineages included lineage B (BK151, BK370, and BK613), CF313, OV700, and YR750; all remaining lineages were treated as non-endosphere. KO frequencies were calculated across all ANI-defined lineages, and enrichment was defined as presence in ≥2 endosphere lineages and in ≤4 of 11 non-endosphere lineages. (**C**) Growth of *Variovorax* sp. CF313 wild-type (WT) and L-fucose pathway mutant (CF313Δfuc) over 72 h in 3-(N-morpholino)propanesulfonic acid (MOPS) minimal medium supplemented with 0.1% glucose or 0.1% L-fucose. (**D**) Root colonization by wild type and CF313Δfuc. Box-and-whisker plots show total CFUs recovered from *Populus* roots. The fucose pathway mutant exhibited significantly reduced colonization (*P* = 0.0022).

Using these lineage definitions, we next compared the KEGG Ortholog (KO) profiles across endosphere- and non-endosphere-associated lineages. A KO was considered enriched if it occurred in at least 2 endosphere lineages and in no more than 4 of 11 non-endosphere lineages, resulting in 131 endosphere-enriched KOs (Fig. 3A; Table S2). The top enriched KOs (Fig. 3B) highlight a set of functions shared across endosphere-associated lineages. Several of the strongest signals were linked to host-derived sugar metabolism, including L-rhamnose mutarotase (K03534) and the L-fucose degradation enzymes K18333 and K18334, which indicate a conserved capacity for utilizing deoxyhexoses abundant in plant tissues. A coordinated set of Type III secretion system proteins (K03219 and K03222 through K03230) was also enriched, consistent with a role for secretion and interaction machinery in endosphere colonization.

Across all 131 enriched KOs, several broad functional themes emerged. Endosphere-associated lineages were enriched in carbohydrate and deoxyhexose utilization; high-affinity nutrient transport for nitrogen, sulfur, and metals; and detoxification pathways such as oxidative stress defenses and metal efflux. Secretion and export systems, including Type III, IV, and VI secretion machinery and ATP-binding cassette (ABC) transporters, were similarly overrepresented. Numerous AraC, LysR, and DeoR regulators and several two-component systems indicated expanded regulatory capacity. Together, these signatures reveal a strategy that integrates metabolic specialization, nutrient acquisition, environmental sensing, and interaction functions that differentiate successful endosphere-colonizing *Variovorax* lineages.

### L-fucose utilization pathway enables endosphere colonization

Several endosphere-enriched KOs belonged to the L-fucose utilization pathway, including L-fucose dehydrogenase (K18333) and L-fuconate dehydratase (K18334) (Fig. 3B). To test whether this pathway contributes to endosphere colonization by strain CF313, we generated a deletion of the L-fuconolactonase gene (PMI12_01353). L-fuconolactonase was selected because it is encoded at the start of the fucose utilization locus in CF313 and initiates the sequence of reactions used by *Variovorax* to process fucose (Fig. S2). The resulting CF313Δfuc strain failed to grow on L-fucose as a sole carbon source, while the wild type showed robust growth under the same conditions (Fig. 3C). When inoculated into sterile *P. deltoides* microcosms, the mutant exhibited significantly reduced colonization compared to wild-type CF313, with a statistically significant decrease in CFUs recovered from root tissue relative to the inoculum (*P* = 0.0022) (Fig. 3D).

Several non-endosphere strains also encoded the complete L-fucose utilization pathway (Fig. S2), raising the question of whether pathway presence alone is predictive of endosphere enrichment. To explore this, we tested all 28 *Variovorax* strains in MOPS minimal medium supplemented with 0.1% L-fucose as the sole carbon source. Three strains, CF313, OV700, and YR750, were able to grow under these conditions, with OD₆₀₀ increasing at least 3-fold over 72 h (Fig. S3). Other strains, which included those with intact fucose pathways, such as BK151, BK613, BK370, and YR634, showed little to no growth. These findings highlight that gene presence does not necessarily imply functional activity and suggest that preferential colonization of the endosphere may depend not only on specific metabolic capabilities but also on how effectively those functions are deployed under host-associated conditions.

### Orthogroup-based profiling reveals reduced and distinct functional capacity in the endosphere

While strain-based analyses can identify dominant endosphere colonizing strains, they may overlook functions encoded by low-abundance strains that nonetheless possess traits relevant to endosphere adaptation. We hypothesized that such traits may be present in additional *Variovorax* strains that were not classified as enriched at the strain level and that examining gene content across the community could help reveal them. To test this, we inferred orthogroups (sets of homologous genes grouped by shared evolutionary origin) from amino acid sequences of all 28 *Variovorax* strains. Metagenomic reads from DefCom-inoculated root compartments were then mapped to each genome and aggregated at the orthogroup level. This allowed us to quantify their relative abundances as counts per million (CPM) and detect orthogroups associated with endosphere enrichment, independent of whether an individual *Variovorax* strain was considered dominant.

**Fig 4:**
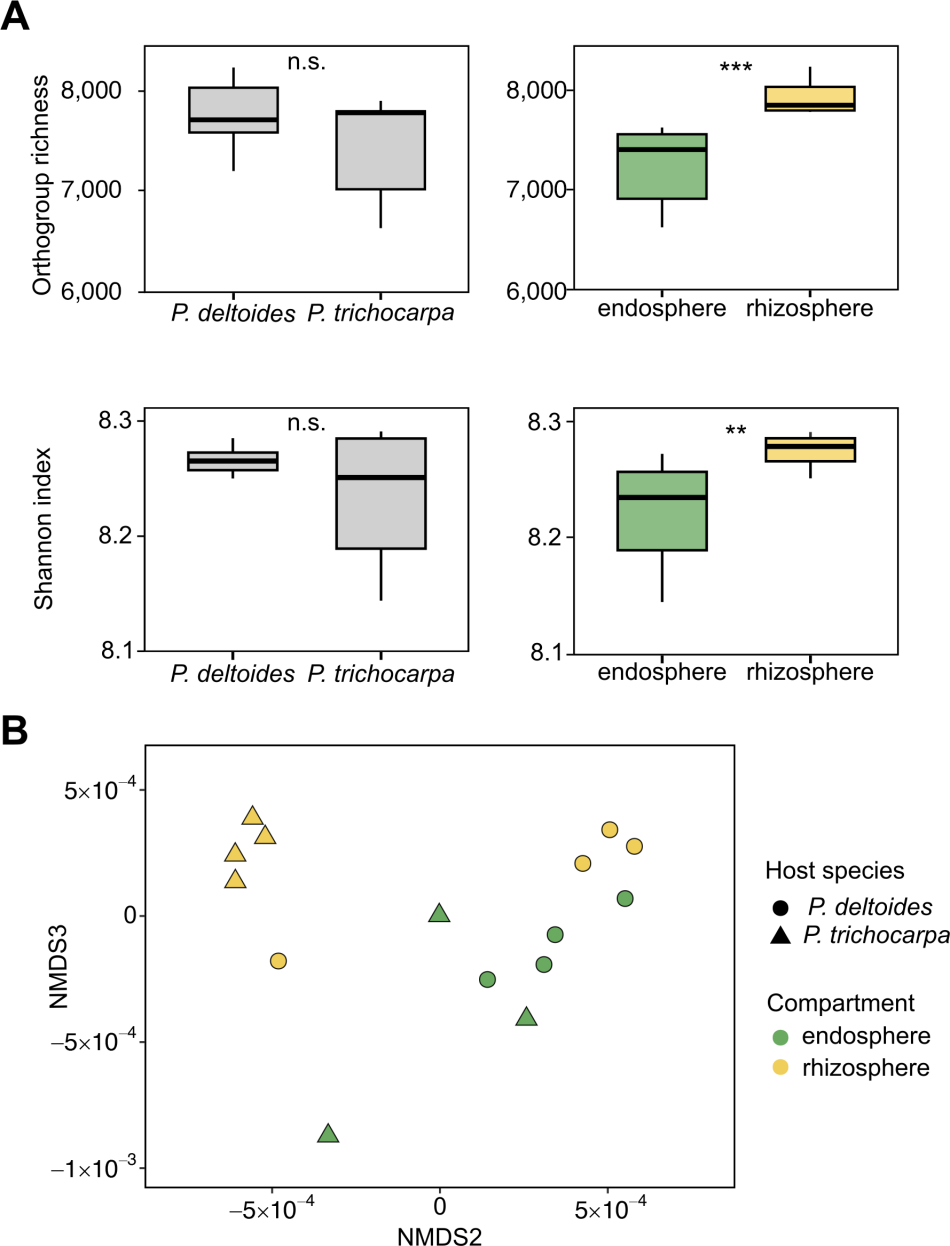
(**A**) Alpha diversity of orthogroup profiles across tree types and compartments. Boxplots show orthogroup richness and Shannon diversity for each tree species (left) and compartment (right). Significance was assessed using ANOVA and Wilcoxon tests; asterisks indicate significant compartment differences (***, **), and “n.s.” denotes non-significant tree species comparisons. (**B**) NMDS ordination of *Variovorax* orthogroup functional profiles based on Bray–Curtis dissimilarities of metagenomic CPM values. Points represent root-associated samples colored by compartment (endosphere vs. rhizosphere) and shaped by tree species (*P. deltoides* vs. *P. trichocarpa*).

To assess the extent to which the *Populus* endosphere selects for a distinct set of microbial traits, we analyzed alpha and beta diversity of orthogroup abundance profiles (expressed as counts per million, CPM) across compartments and host species. The endosphere exhibited significantly reduced richness and evenness in orthogroup profiles compared to the rhizosphere (ANOVA and Wilcoxon tests; *P* < 0.05) (Fig. 4A), consistent with selective filtering for a narrower set of traits. Non-metric multidimensional scaling (NMDS) ordination of Bray–Curtis dissimilarities revealed clear clustering by root compartment and host species, with both factors contributing significantly to variation in orthogroup composition (PERMANOVA; *P* < 0.05) (Fig. 4B). Compartment explained 21% of the variance, and host species explained 14%. These results indicate that specific orthogroups differ in abundance between compartments, suggesting that the endosphere imposes selective constraints on community functional potential.

### Differential abundance analysis identifies conserved endosphere-enriched orthogroups

**Fig 5:**
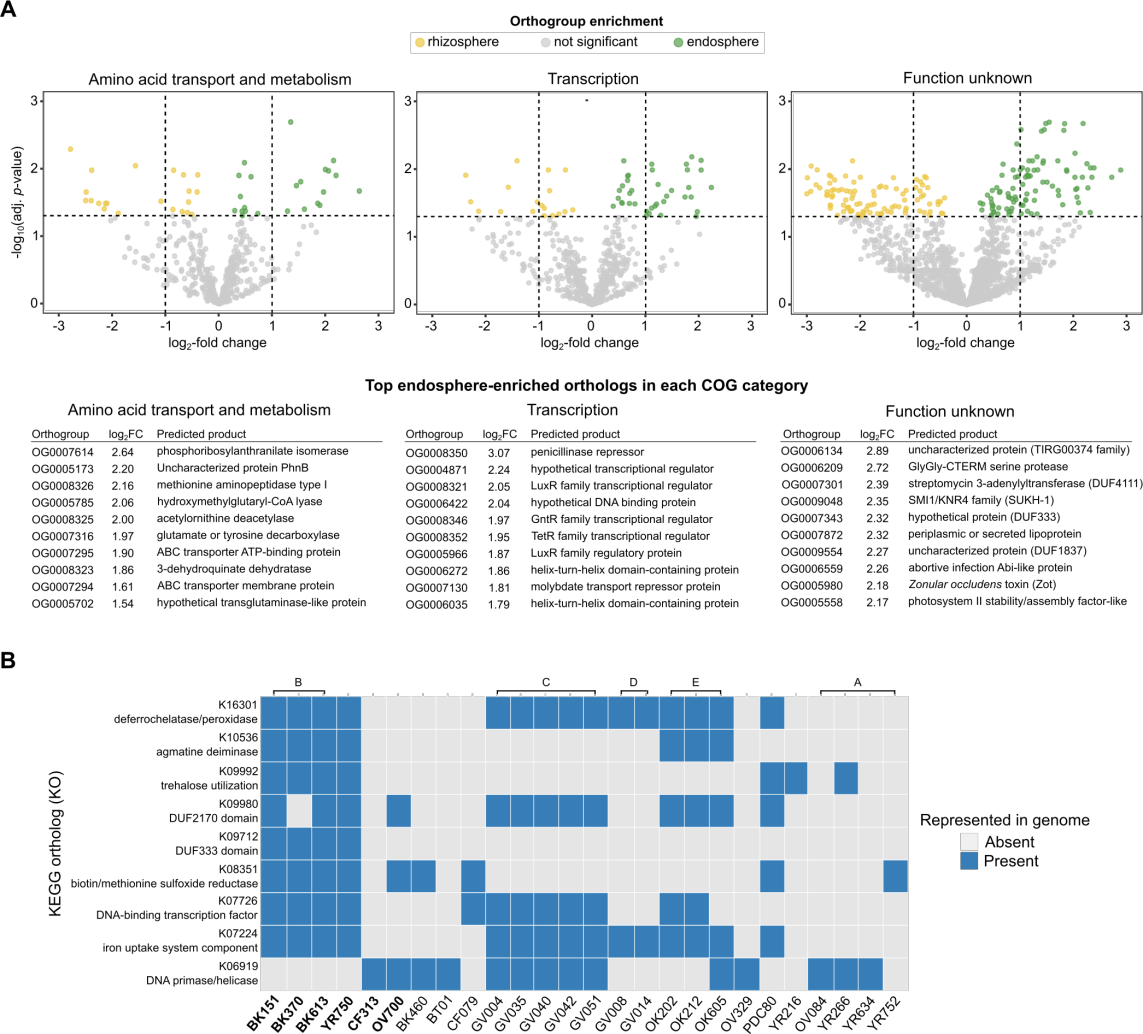
(**A**) Differential representation of orthogroups across COG functional categories in endosphere and rhizosphere compartments. Volcano plots show log₂ fold change (endosphere vs. rhizosphere) versus adjusted *P*-value from limma-voom analyses of raw orthogroup counts. Orthogroups enriched in the endosphere (log₂FC > 0, FDR < 0.05) are shown in green, those enriched in the rhizosphere (log₂FC < 0, FDR < 0.05) in yellow, and non-significant orthogroups in gray. Vertical dashed lines indicate log₂FC thresholds of ±1, and the horizontal line, the FDR threshold of 0.05. The 10 most enriched orthogroups in each COG category in order by log₂FC are listed. (**B**) Comparison of KOs enriched across analysis frameworks. The heatmap displays the presence of nine KOs identified as significantly enriched in the endosphere by three statistical methods (DESeq2, limma-voom, and metagenomeSeq) and that were more frequent in ANI-defined endosphere-associated lineages. Rows represent *Variovorax* strains (endosphere-enriched strains in bold), and columns are grouped by ANI-defined lineages indicated by brackets above the heatmap. Strains that lacked all nine KOs are excluded.

To identify the gene groups consistently associated with endosphere enrichment, we first aggregated all detected orthogroups across the community, regardless of their strain of origin, and compared their normalized read abundances between endosphere and rhizosphere metagenomes. Statistical enrichment of orthogroups in the endosphere was then assessed using multiple complementary approaches, including DESeq2 (using raw counts), limma-voom (with per-copy normalization), and metagenomeSeq (zero-inflated modeling with CSS normalization). This conservative strategy yielded 249 orthogroups consistently enriched in the endosphere across all three methods (Fig. S1, Table S3). These orthogroups included genes annotated as ABC transporters, superoxide dismutases, and transcriptional regulators (LysR, AraC, and TetR families). Functional annotation of these orthogroups using Clusters of Orthologous Group (COG) categories showed that transcription, amino acid transport and metabolism, and functions of unknown category contained many endosphere-enriched orthogroups (Fig. 5A). Additional enriched categories included signal transduction and inorganic ion transport and metabolism (Fig. S5).

To place these orthogroup patterns in a broader functional context, we focused on KOs that were enriched both in the orthogroup-level metagenomic analysis and the earlier lineage-level KO frequency analysis of endosphere-enriched functions (Fig. 5B). This intersection yielded a compact set of nine functions spanning DNA replication (K06919), transcriptional regulation (K07726), iron uptake (K07224 and K16301), stress response and redox metabolism (K08351), carbohydrate utilization (K09992), nitrogen metabolism (K10536), and two conserved domains of unknown function (K09712 and K09980). These functions were consistently found in the dominant endosphere lineage B (BK151, BK370, and BK613) and in YR750, with sporadic representation in a subset of non-endosphere strains. The strongest lineage specificity was observed for K09712 (DUF333), which was restricted to lineage B and YR750 and never detected in strains lacking endosphere enrichment. In contrast, iron-uptake (K07224 and K16301), stress-response (K08351), and carbohydrate-utilization functions (K09992) were enriched in endosphere-associated lineages but were not exclusive to them. Collectively, this set of nine KOs points to a recurrent combination of regulatory, nutrient-acquisition, redox, and carbohydrate-metabolism functions that distinguishes successful endosphere colonizers from the broader *Variovorax* pool.

### Endosphere-enriched orthogroups suggest divergent colonization strategies when bacteria are in different *Populus* hosts

**Fig 6:**
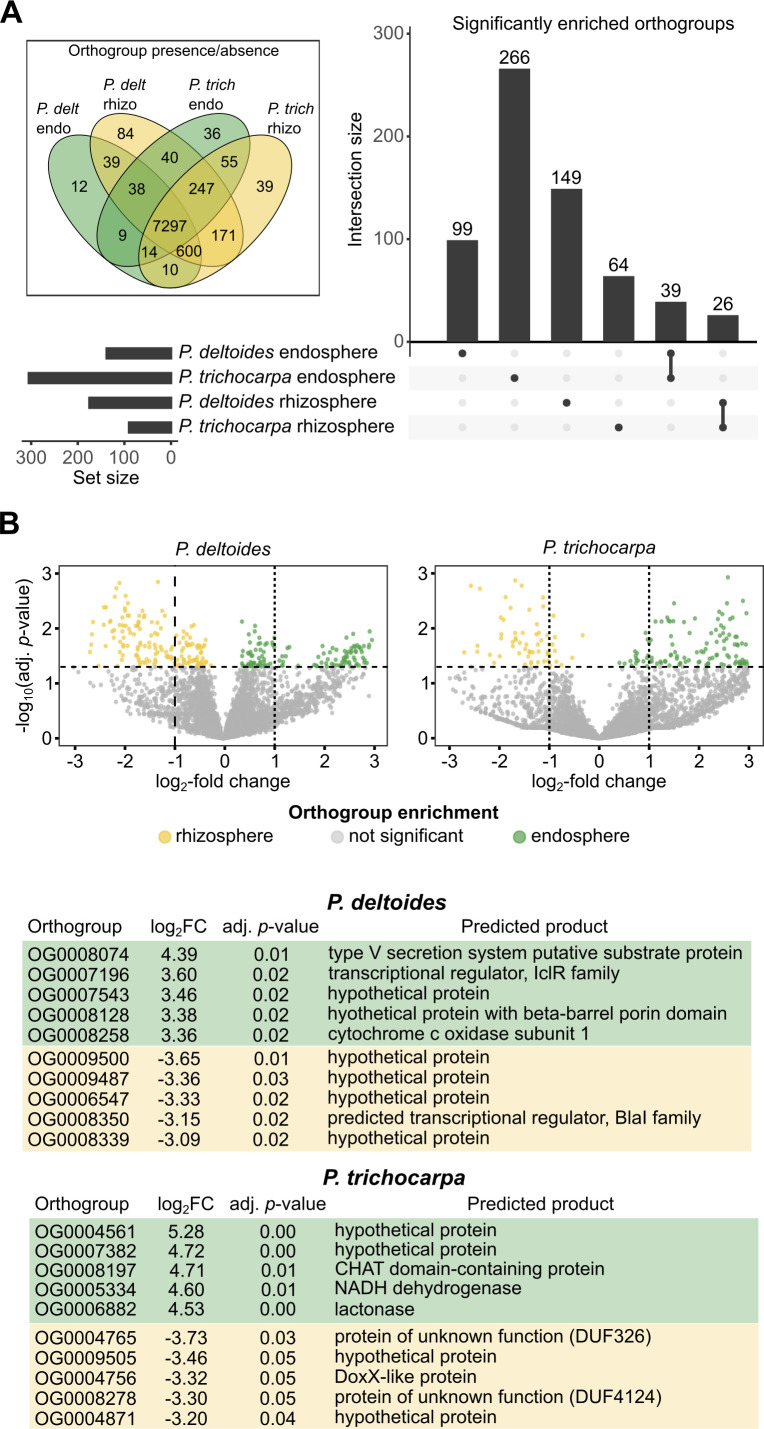
(**A**) UpSet plot showing significantly enriched orthogroups identified by limma-voom analysis of endosphere versus rhizosphere metagenomes for each *Populus* host species. Analyses were conducted separately for *P. deltoides* and *P. trichocarpa* to identify orthogroups enriched within each host; shared enrichments represent functions independently significant in both. Inset: Venn diagram of orthogroups detected (presence/absence) in *P. deltoides* (*P. delt*) and *P. trichocarpa* (*P. trich*) endosphere (endo) and rhizosphere (rhizo) metagenomes. The inset depicts all orthogroups found in each tree × compartment combination, including those identified as not significantly enriched. (**B**) Top significant orthogroups enriched in the endosphere (green) or rhizosphere (yellow) of *P. deltoides* and *P. trichocarpa* based on log_2_ fold-change estimates from limma-voom.

Endosphere-enriched orthogroups showed host-specific patterns, suggesting divergent colonization strategies between *P. deltoides* and *P. trichocarpa* (Fig. 6). Differential abundance analyses were performed separately for each host using limma-voom, comparing endosphere and rhizosphere metagenomes within each species. Orthogroups significantly enriched in each host were then compared to identify functions unique to or shared between hosts. In *P. deltoides*, enriched functions were biased toward host interaction and membrane remodeling, such as type V secretion system substrates, outer membrane autotransporters, and patatin-like phospholipases, as well as redox-related enzymes like multicopper oxidases and NADPH:quinone reductases. These suggest a colonization strategy shaped by direct engagement with host surfaces and oxidative stress mitigation. In contrast, *P. trichocarpa* favored specialized metabolic functions, including aromatic compound degradation, nitrogen assimilation, and sulfur metabolism. A set of 39 orthogroups was consistently enriched in both hosts, largely comprising core functions such as central carbon metabolism (e.g., glutamate dehydrogenase and succinyl-CoA synthetase), transporters, proteases, and diverse transcriptional regulators (Table S4). Together, these patterns suggest that while both hosts select for a common set of metabolic and regulatory functions, their distinct enrichment signatures reflect different physiological filters shaping endosphere colonization.

## DISCUSSION

*Variovorax* is consistently enriched in the *Populus* rhizosphere; however, only a subset of strains succeeds in colonizing the endosphere. Because all strains in our experiment were originally isolated from *Populus* roots or rhizospheres and introduced at equal starting abundance, their relative enrichment in the endosphere reflects compartmental specialization among naturally *Populus*-associated strains rather than absolute colonization potential. Endosphere colonization is a multistep ecological challenge requiring microbial access to the root, competition within the rhizosphere, survival of the chemically selective internal environment, and the capacity to engage the host in functionally meaningful ways (29, 30). Our strain-resolved metagenomic analyses confirm that endophytes represent a selected subset of the broader rhizosphere community, and by resolving strain-level contributions, our analysis reveals fine-scale functional variation that is often masked in OTU-based or bulk metagenomic data. This approach shows that ecological filtering leaves reproducible genomic signatures that can illuminate the traits necessary for successful endosphere colonization within *Populus*.

Endosphere colonization outcomes among *Variovorax* strains are shaped less by genomic potential than by how those functions are regulated and expressed within the host. In our defined community, experimental results showed that related strains exhibited different patterns of enrichment between root compartments. Comparative phylogenetic and ANI analyses reinforce this observation, showing that dominant endosphere colonizers occur across both closely related and more divergent lineages. The presence of successful colonizers throughout the *Variovorax* tree indicates that endosphere enrichment can occur both within closely related clades and independently across more diverse lineages. This pattern suggests that endosphere competence is further shaped by regulatory and ecological factors that extend beyond phylogenetic boundaries. Supporting this distinction, the conservation of canonical plant-beneficial genes such as ACC deaminase and IAA degradation (31) across all *Variovorax* genomes underscores that these broadly distributed traits are not sufficient to explain preferential colonization of the endosphere. Instead, the orthogroups enriched in the endosphere were dominated by transcriptional regulators and other regulatory functions, suggesting that dynamic control of gene expression, rather than static gene inventories, is central to colonization of the endosphere.

Our results reveal two ecological strategies for endosphere colonization: metabolic competitiveness and persistence through regulatory adaptation. Strains that reached high abundance and grew robustly on fucose exemplify rapid resource capture, underscoring competition for host-derived carbon as a major driver of success, a pattern consistent with synthetic community studies of *Populus* rhizosphere bacteria (32, 33). In contrast, strains that did not grow on fucose despite possessing the pathway were instead enriched in functions drawn from the consensus set of nine endosphere-associated KOs, including iron-uptake systems, redox and stress-response enzymes, nitrogen and trehalose metabolism, and regulatory proteins. These traits reflect a strategy centered on maintaining cellular homeostasis and adjusting gene expression within the chemically selective host environment (34–37). Flexible colonizers such as YR750 encoded both fucose utilization and the regulatory traits found in persistence-oriented strains, suggesting the capacity to use multiple strategies depending on environmental conditions. Taken together, these outcomes show that endosphere persistence can arise through distinct ecological routes, either dominance via rapid substrate use or endurance through regulatory control, and that success is not determined by genomic content alone but by how strains deploy their traits in the host environment.

Host species further shaped which traits were favored, indicating that *Populus* species impose distinct selective filters on their microbial partners. *P. deltoides* endophytes were biased toward redox-active enzymes and membrane remodeling functions, while *P. trichocarpa* endophytes favored transcriptional regulators and amino acid metabolism. These distinctions underscore that genomic signatures of colonization are not uniform across hosts but are contingent on plant context. More broadly, differences in the traits favored by *Populus* species align with emerging evidence that endosphere colonization in long-lived woody hosts may operate through mechanisms distinct from those described in annual crops and model plants, where colonization is often framed through pathogen-associated secretion and invasion systems (38–40). While not directly tested here, our results may help clarify this distinction by revealing regulatory and metabolic traits linked to stable associations in tree endophytes.

Beyond host genotype, the ecological context in which colonization occurs also shapes microbial success. Fucose, a major component of plant cell-wall polysaccharides and a prominent sugar in root mucilage at the growing root tip (41–43), may become available to microbes through root cell turnover or the release of soluble carbohydrates in root exudates, which contain sugars and other primary metabolites (44). Although we did not quantify fucose concentrations, host-specific differences in carbohydrate availability and utilization could help explain variation in colonization patterns across *Populus* species. In natural soils, these resource-driven interactions occur within the broader microbiome, where metabolic competition, antagonism, and cooperation among community members further influence which strains gain access to the endosphere. Together, these factors highlight that colonization outcomes are emergent properties of both host filtering and community ecology and that predictive models of root microbiome assembly must integrate metabolic, regulatory, and ecological dimensions.

Our results demonstrate that *Variovorax* colonization of the *Populus* endosphere is governed by strategies of metabolic competitiveness, regulatory adaptation, or a combination of both and that host species further influences which traits are successful. While stochastic influences cannot be completely excluded, the reproducible enrichment of specific traits across replicates and host species indicates that selection is the main force shaping colonization outcomes. Enriched genes highlight ecological differentiation; however, gene content alone does not fully explain colonization outcomes, emphasizing the importance of regulatory control and host context. Future work integrating genomic profiles with dynamic transcriptomic and proteomic measurements, coupled with predictive modeling, will be essential to determine how colonization strategies are deployed under native conditions. Ultimately, this genome-resolved perspective advances mechanistic understanding of niche-specific root microbiome assembly and informs efforts to design microbial consortia that promote plant health and resilience across variable environments.

## MATERIALS AND METHODS

### Genome collection and phylogenetic analysis

Genomes of 28 *Variovorax* strains previously isolated (19) from the rhizosphere and endosphere of *P. deltoides* and *P. trichocarpa* were obtained from the Integrated Microbial Genomes & Microbiomes (IMG/M) database (45) (Table S1). The two- or three-letter prefix in each strain name corresponds to the original isolation site of that strain. Single copy orthologs (*N* = 1,447) were identified using Broccoli v1.2 (46), and coding sequences were aligned using translator v1.1-2 (47) and MAFFT v7.508 (48). Alignments were trimmed using ClipKIT v1.3.0 (49) to retain sites with less than 30% gaps and then concatenated. Phylogenetic reconstruction was performed using IQ-TREE2 v2.2.0.3 (50) using ModelFinder (51) to perform model selection and determine the best partitioning scheme. Tree topology support was evaluated using the ultrafast bootstrap approximation with 1,000 bootstrap replicates (52). Pairwise ANI was calculated using the fastANI (53) module implemented in KBase (54) to assess species-level relatedness among the 28 genomes.

### Bacterial defined community (DefCom) assembly and preparation

All bacterial strains were streaked from frozen glycerol stocks to rich medium plates (Reasoner’s 2A [R2A], Franklin Lakes, BD Difco, NJ, USA) (55). A single colony from each plate was used to inoculate 5 mL of R2A and incubated overnight at 30°C and 200 rpm by continually shaking. Once turbid, bacterial suspensions were pelleted, washed with sterile water to remove remaining media, and then diluted with sterile water to an OD600 = 0.01. Equal volumes of each OD-normalized strain culture were combined into a single DefCom inoculum, and 10 mL of the DefCom inoculum was used to inoculate soil for plant experiments.

### Plant inoculation and growth

Axenic *P. deltoides* (genotype WV94) and *P. trichocarpa* (genotype BESC-819) shoots were generated from surface-sterilized tissue-culture cuttings and maintained under sterile conditions following a standard procedure (56). Rooted shoots were transplanted into sterile polycarbonate vessels (3 × 3 × 4″) containing 120 cm³ of inert clay (Pro’s Choice Rapid Dry, Alpharetta, GA) supplemented with 80 mL of 1× Modified Hoagland’s solution. Twelve DefCom-inoculated and 12 uninoculated control replicates were prepared for each tree species. Immediately prior to transplanting, 10 mL of DefCom inoculum was added to the clay and mixed thoroughly for 30 s. Microcosms were sealed and maintained under controlled growth chamber conditions (16 h photoperiod, 21°C/17°C day/night, 60% RH) for 3 weeks. Uninoculated controls remained sterile, confirmed by plating rinse fractions on R2A medium.

### Root colonization

After harvest, roots were gently washed twice by submerging in sterile deionized water (20 mL each) to remove adhering clay; wash fractions were collected for rhizosphere analysis. Roots were then placed in 15 mL sterile PBS and sonicated for 30 s at 50–60 Hz. The buffer was collected as the rhizoplane fraction. This sonication step was repeated twice with fresh PBS, after which the washed roots were designated as the endosphere fraction and used for downstream enrichment. Following the final sonication, roots were designated as the endosphere fraction and prepared for downstream enrichment using a protocol adapted from Pelletier and coworkers (57).

An ethanol- and UV-sterilized (15 min) grinder (KSM2; Braun, Kronberg, Germany) was used to disrupt and homogenize the endosphere roots in 20 mL sterile Milli-Q. The homogenate was poured through sterile miracloth and collected in a 50-mL conical tube. The endosphere homogenates were centrifuged at 500 × *g* for 5 min at 10°C (Spinchron R; Beckman Coulter, Brea, CA). The supernatants were transferred to new conical tubes and centrifuged at 5,500 × *g* for 20 min at 10°C (Sorvall Evolution RC; Thermo Scientific, Carlsbad, CA). The supernatants were discarded, and the pellet was resuspended in 20 mL of bacterial cell extraction (BCE) buffer (50 mM Tris-HCl [pH 7.5], 1% Triton X-100, 2 mM 2-mercaptoethanol). The suspension was filtered through sterile miracloth and transferred to a sterile 50-mL Oak Ridge tube (Nalgene, Rochester, NY). The suspensions were centrifuged at 10,000 × *g* for 10 min at 10°C, and the supernatants were discarded. The pellet was resuspended in 20 mL BCE buffer, filtered again through sterile miracloth, and centrifuged at 10,000 × *g* for 10 min at 10°C. The supernatant was discarded, and the pellet was resuspended in 1 mL of 50 mM Tris-HCl (pH 7.5). The suspension was centrifuged at 10,000 × *g* for 3 min, the supernatant removed, and the pellet resuspended in 1 mL of 50 mM Tris-HCl (pH 7.5). The final pellet was stored at –20°C for DNA extraction.

### DNA extraction and metagenomic sequencing

DNA from the four endosphere and rhizosphere samples was extracted using the DNeasy Plant Pro Kit (Qiagen, Venlo, the Netherlands) according to the manufacturer’s instructions for each DefCom/*Populus* species. Library preparation was performed using the Nextera XT DNA Library Preparation Kit (Illumina), following the manufacturer’s protocol. Prepared libraries were quantified using the Qubit dsDNA BR Assay Kit (Invitrogen, Carlsbad, CA, USA), and quality was assessed on an Agilent 2100 Bioanalyzer using a DNA 7500 Chip Kit (Agilent Technologies, Santa Clara, CA, USA). Libraries were pooled at equal molar concentrations and paired-end sequenced (2 × 251 bp) on an Illumina MiSeq instrument with v2 chemistry.

### Strain abundance and differential enrichment analysis

Reads were demultiplexed on the MiSeq and processed using Atropos v1.1.28 (58) for adapter trimming and quality filtering (q = 15, length < 150). To exclude host-derived DNA sequences from the differential enrichment analysis, filtered reads were aligned to the appropriate *Populus* genome, either *P. trichocarpa* (Phytozome genome ID: 444, NCBI taxonomy ID: 3694) (59) or *P. deltoides* (Phytozome genome ID: 445; NCBI taxonomy ID: 3696) (60). Reads that were not mapped onto the *Populus* genome were imported into R (v4.0.2) and were mapped to all DefCom *Variovorax* genomes using Rsubread v2.2.4 (61), allowing up to 16 equally best mapping locations (nBestLocations = 16). To minimize double-counting among closely related strains (>98% ANI), features were counted using unique-mapping mode (countMultiMappingReads = FALSE), which excludes reads that map equally well to multiple reference genomes. For comparison, a separate analysis using fractional assignment of multi-mapped reads (countMultiMappingReads = TRUE, fraction = TRUE) was performed to evaluate the robustness of enrichment patterns. Overall enrichment patterns were consistent across mapping strategies, and any inconsistencies were limited to strains sharing ≥99% nucleotide identity. All subsequent analyses and figures use the unique-mapping counts.

Read counts were normalized using TMM implemented in edgeR v3.30.3 (62). Voom (63) was used to transform count data to log2-counts per million (logCPM), estimate the mean-variance relationship, and use this to compute appropriate observational-level weights. Contrasts of plant species, plant compartment, and their interaction were carried out using limma v3.44.3 (64) with Benjamini-Hochberg correction for multiple testing. Log₂ fold changes (endosphere vs. rhizosphere) were used to quantify differential compartment association rather than absolute colonization efficiency, since all strains were inoculated at equal initial abundance.

### Functional enrichment analysis (KO/COG)

Functional annotation of all 28 *Variovorax* genomes was performed using EggNOG-mapper v2 (65, 66) to assign KOs, COGs, and additional functional categories. Strains were first grouped into ANI-defined lineages using a ≥97% ANI threshold, and lineages containing at least one dominant endosphere colonizer were classified as endosphere-associated lineages. For each KO, presence within a lineage was scored as 1 if the KO occurred in any genome in that lineage and 0 otherwise, and KO frequency was calculated as the proportion of lineages in each group (endosphere or non-endosphere) that contained the annotation. A KO was considered endosphere-enriched if it occurred in at least 2 endosphere-associated lineages and in no more than 4 of 11 non-endosphere lineages. This lineage-based framework standardized functional comparisons across genomes and enabled subsequent enrichment analyses and visualization of trait distributions at both the lineage and orthogroup levels.

### Mutant construction and validation

Flanking regions of approximately 500 bp were amplified on either side of the L-fuconolactonase gene (PMI12_01353) using primers with homologous overlaps. All primers for cloning were designed using the NEBuilder design tool (New England Biolabs (NEB), Ipswitch, MA), and the oligos were synthesized by Integrated DNA Technologies (Integrated DNA Technologies (IDT), Coralville, IA) or Eurofins Genomics (Eurofins Genomics, Louisville, KY). Gibson Assembly Master Mix (NEB) was then used according to the manufacturer’s protocol to clone the amplified fragments into pMO130 suicide vector backbone (Addgene plasmid #27388; http://n2t.net/addgene: 27388; RRID:Addgene_27388), which carries kanamycin resistance, and the *sacB* gene, which confers sucrose sensitivity (67). The assembled plasmid was transformed into NEB 5-alpha (NEB) *Escherichia coli* cells by heat shock. The plasmid construct was then purified using a miniprep kit (Qiagen, Valencia, CA) and transformed by electroporation into the conjugative *E. coli* strain WM3064 (68). Mating with *Variovorax* strain CF313 was accomplished by mixing washed donor and recipient cells at a 1:10 ratio, concentrating by centrifugation, and depositing onto a sterile filter on an R2A plate. These were allowed to mate overnight at 30°C. Cells were subsequently washed from the filter and plated on R2A medium supplemented with 50 μg/mL kanamycin sulfate to select for plasmid-integrated CF313. One resulting kanamycin-resistant colony was grown overnight in liquid R2A at 30°C with 200 rpm shaking without selection to allow for final chromosomal integration to occur. An overnight culture (10 μL) was streaked to single colonies on R2A medium with 10% sucrose for counterselection. Several resulting colonies were then patched onto R2A media and screened using PCR.

### Growth curves

Overnight R2A cultures were pelleted, washed, and resuspended in filter-sterilized 1× MOPS minimal medium supplemented with either 0.1% glucose or 0.1% L-fucose. In 96-well plates, 150 μL of medium and 2 μL of inoculum (adjusted to an initial OD₆₀₀ = 0.01) were combined. Parallel cultures were also grown in R2A medium on the same plate for comparison. OD₆₀₀ measurements were recorded every 10 min for 72 h at 30°C with orbital shaking using a microplate spectrophotometer. Each condition was assayed in triplicate technical replicates.

### Plant colonization by wild-type and mutant strains

Bacterial inoculants were prepared using overnight cultures of CF313 and CF313Δ*fuc* diluted in sterile water to an OD₆₀₀ of 0.01. Individual strains or combined strains (10 mL) were thoroughly mixed into sterile inert clay supplemented with 1× Hoagland’s solution in a microcosm. Newly rooted axenic tissue culture plants of *P. deltoides* (genotype WV94) were individually established in each microcosm and maintained in a controlled-condition growth chamber (16 h photoperiod, 21°C) for 3 weeks.

Plants were gently removed from the boxes in a sterile hood, and roots were dipped several times in sterile water to remove clay and then patted dry with sterile paper towels. After recording root weights, roots were sonicated for 3 min in 15 mL of 0.1× PBS buffer. The buffer was decanted, and the wash was repeated three times with fresh buffer. Washed roots were resuspended in 10 mL 0.1× PBS and homogenized using a Geno/Grinder (SPEX Sample Prep). Homogenates were briefly centrifuged to pellet large debris, and a 10-fold serial dilution was prepared in 0.1× PBS up to 10⁻⁷. For each dilution, 10 μL was spotted in triplicate onto MOPS minimal medium plates supplemented with either 0.1% glucose or 0.1% L-fucose, and plates were allowed to dry in a sterile hood. Colonies were counted after visible growth, and CFUs per gram of root tissue were calculated based on the dilution factor and plated volumes.

### Orthogroup analysis

OrthoFinder (v3.0.1b1) (69) was used to infer orthogroups from amino acid sequences of all 28 *Variovorax* genomes. Functional annotation of all coding sequences was performed with EggNOG-mapper v2 (65, 66), providing KOs, COGs, and additional functional assignments, which were then associated with their respective orthogroups. Metagenomic reads were mapped to the orthogroups, and counts were normalized to counts per million (CPM) to quantify functional abundance. Orthogroup alpha and beta diversity (Bray–Curtis) was visualized using NMDS (vegan package in R). Enriched orthogroups were identified using DESeq2 (70), limma-voom (63), and metagenomeSeq (71), and significance required agreement across all three methods (FDR < 0.05).

## Data Availability

Raw metagenomic sequencing reads have been deposited in the NCBI Sequence Read Archive under BioProject accession number PRJNA1322484. The R code for preprocessing, analysis, and visualization is available at doi: https://doi.org/10.5281/zenodo.17466836.

## SUPPLEMENTAL MATERIAL

Supplemental figures msystems.01605-25-s0001.docx Figures S1 to S5.

Supplemental tables msystems.01605-25-s0002.xlsx Tables S1 to S4.

ASM does not own the copyrights to Supplemental Material that may be linked to, or accessed through, an article. The authors have granted ASM a non-exclusive, world-wide license to publish the Supplemental Material files. Please contact the corresponding author directly for reuse.
