# Supplementary material for: Genomic signatures in Variovorax enabling colonization of the Populus endosphere
Source: mSystems. 2026 Jan 16;11(2):e01605-25. doi: 10.1128/msystems.01605-25 (PMC12911387; doi:10.1128/msystems.01605-25)
Supplement: Supplemental figures — Figures S1 to S5. [file msystems.01605-25-s0001.docx]

**Supplemental figures for “Genomic signatures in *Variovorax* enabling colonization of the *Populus* endosphere”**

**Authors:** Delaney G. Beals*, Dana L. Carper*, Leah H. Hochanadel, Sara S. Jawdy, Dawn M. Klingeman, Bryan T. Piatkowski, David J. Weston, Mitchel J. Doktycz, and Dale. A Pelletier

**
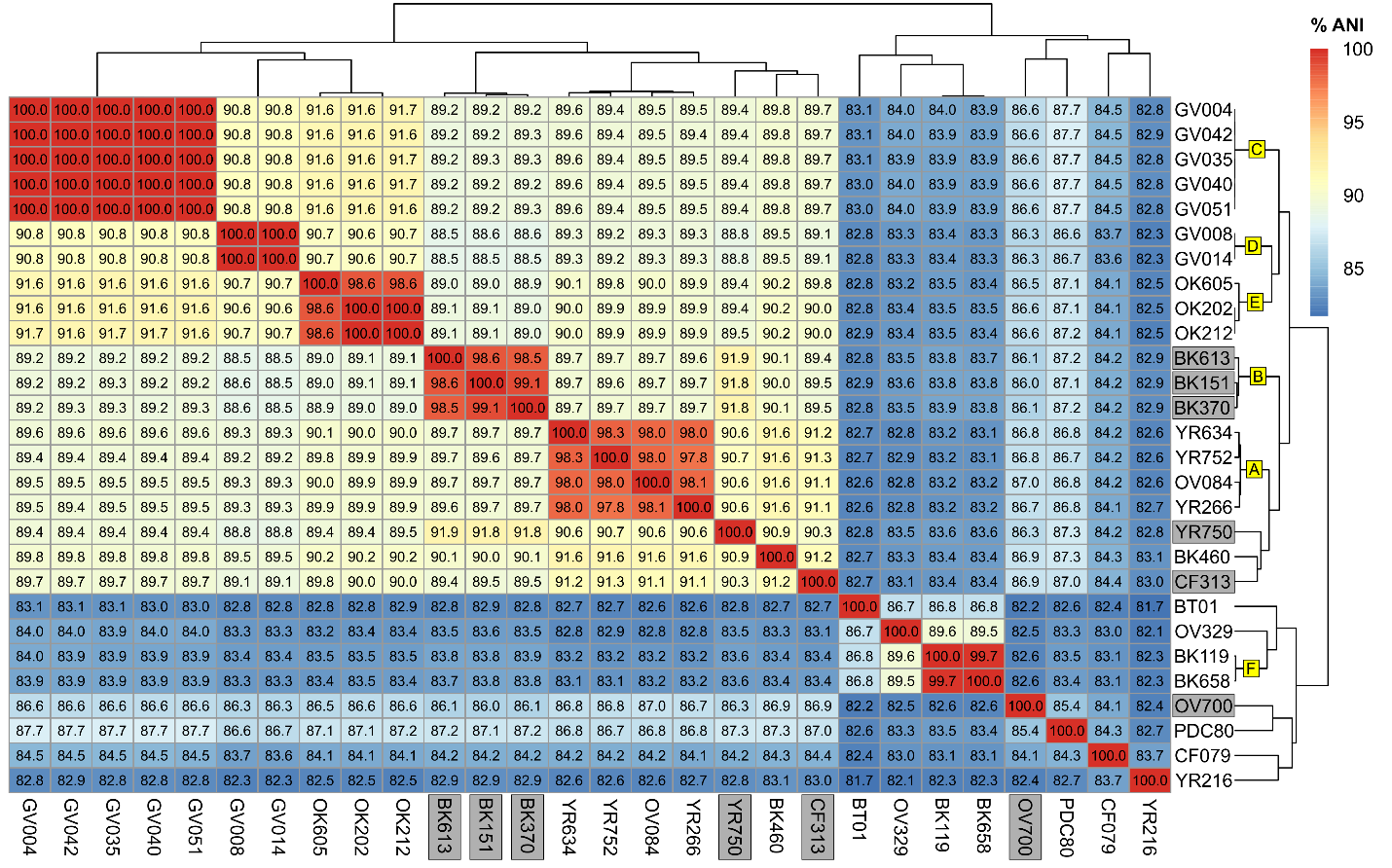
**

**Figure S1**. Pairwise average nucleotide identity (ANI) among the 28 *Variovorax* strains used in this study. ANI values (%) were calculated using FastANI and are displayed as a heatmap, with strains ordered by hierarchical clustering based on pairwise similarity. Gray boxes highlight the six dominant endosphere-colonizing strains identified in the DefCom community. Shared *Variovorax* lineages, defined as groups of strains with ≥97% ANI, are indicated by boxed letter labels along the right dendrogram.


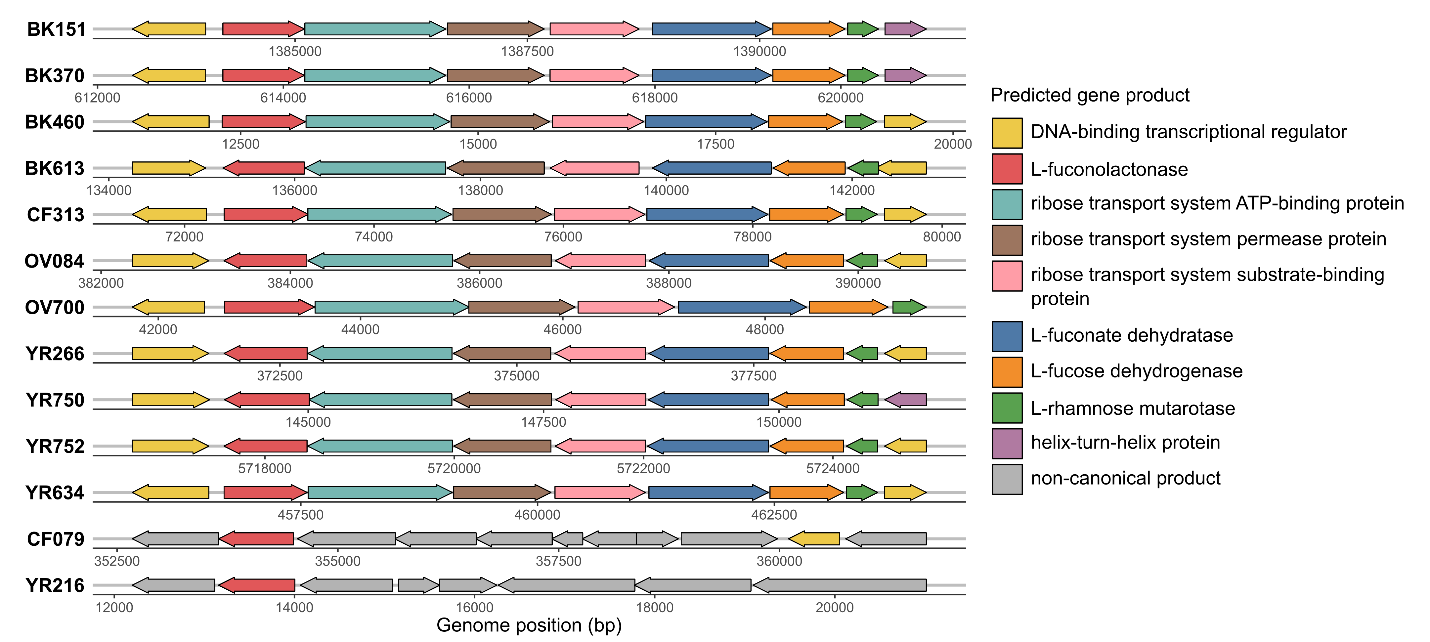


**Figure S2**. Gene‐neighborhood comparisons of the L‑fucose utilization pathway in the 13 *Variovorax* strains (out of 28 genomes screened) that encode KEGG orthologs K18333 (L‑fucose dehydrogenase) and/or K07046 (L‑fuconolactonase).


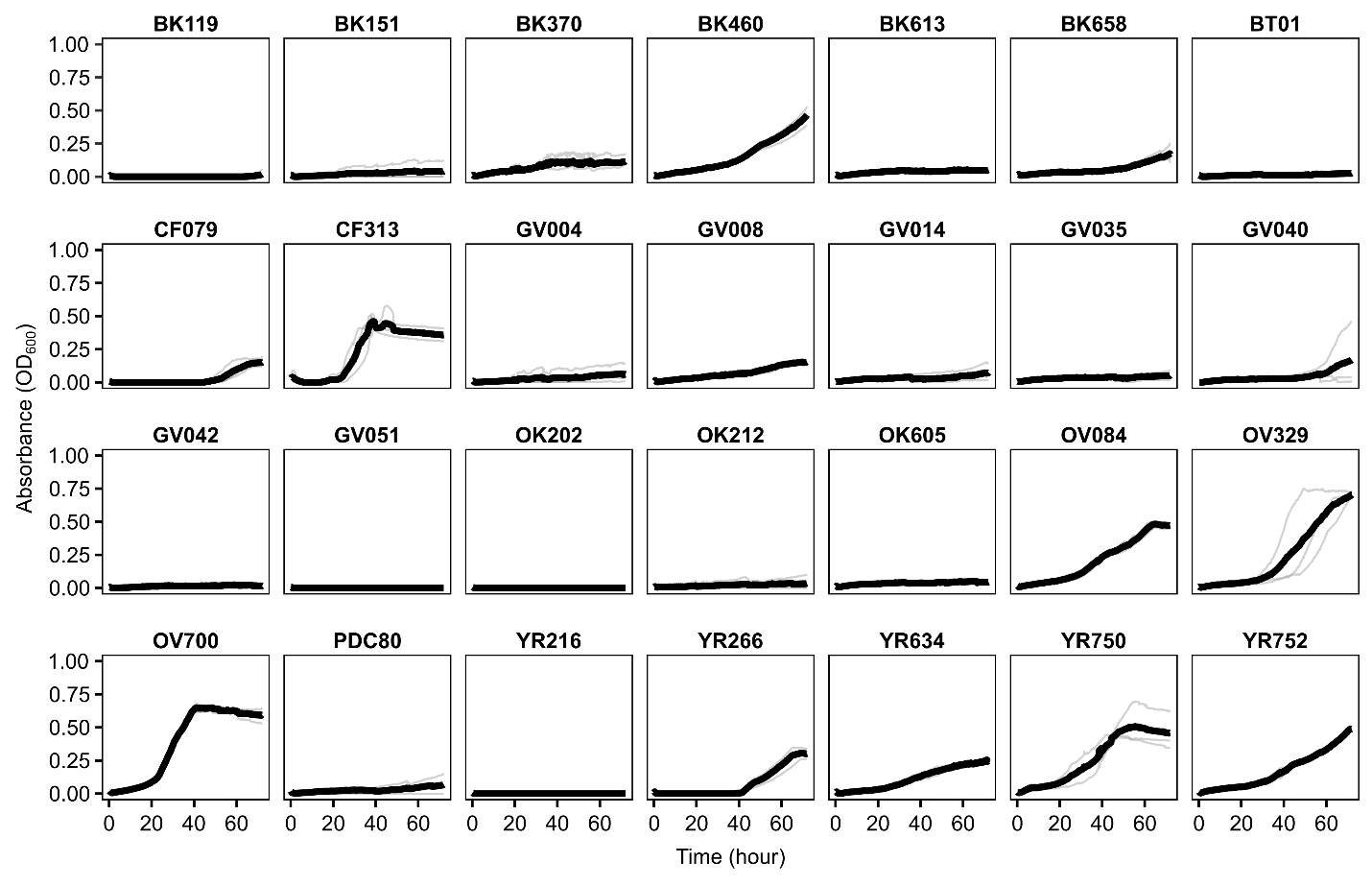


**Figure S3.** Growth of endosphere-enriched *Variovorax* strains on L-fucose. OD₆₀₀ over time for six dominant endosphere strains grown in MOPS minimal medium with 0.1% L-fucose at 25 °C. Lines represent three independent replicates (thin) and the mean (thick).


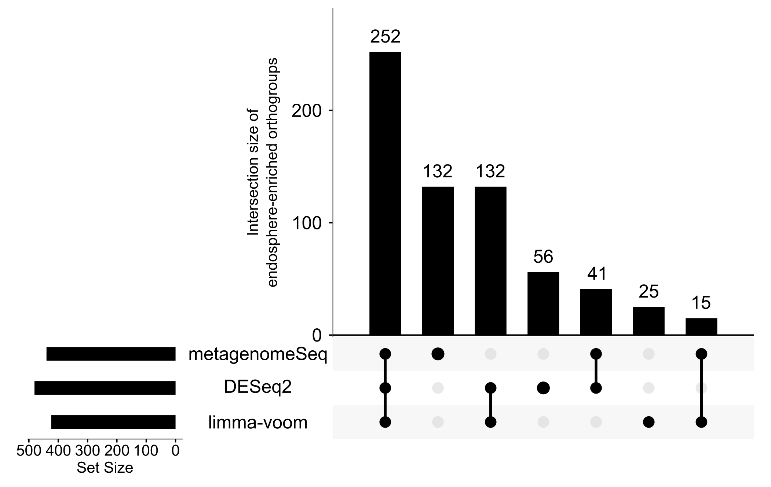


**Figure S4.** Overlap of endosphere-enriched orthogroups identified by DESeq2 (raw counts), limma-voom (TMM normalization with voom transformation), and metagenomeSeq (CSS normalization). Significance was defined as adjusted p < 0.05 and log₂ fold-change > 0 (endosphere > rhizosphere).


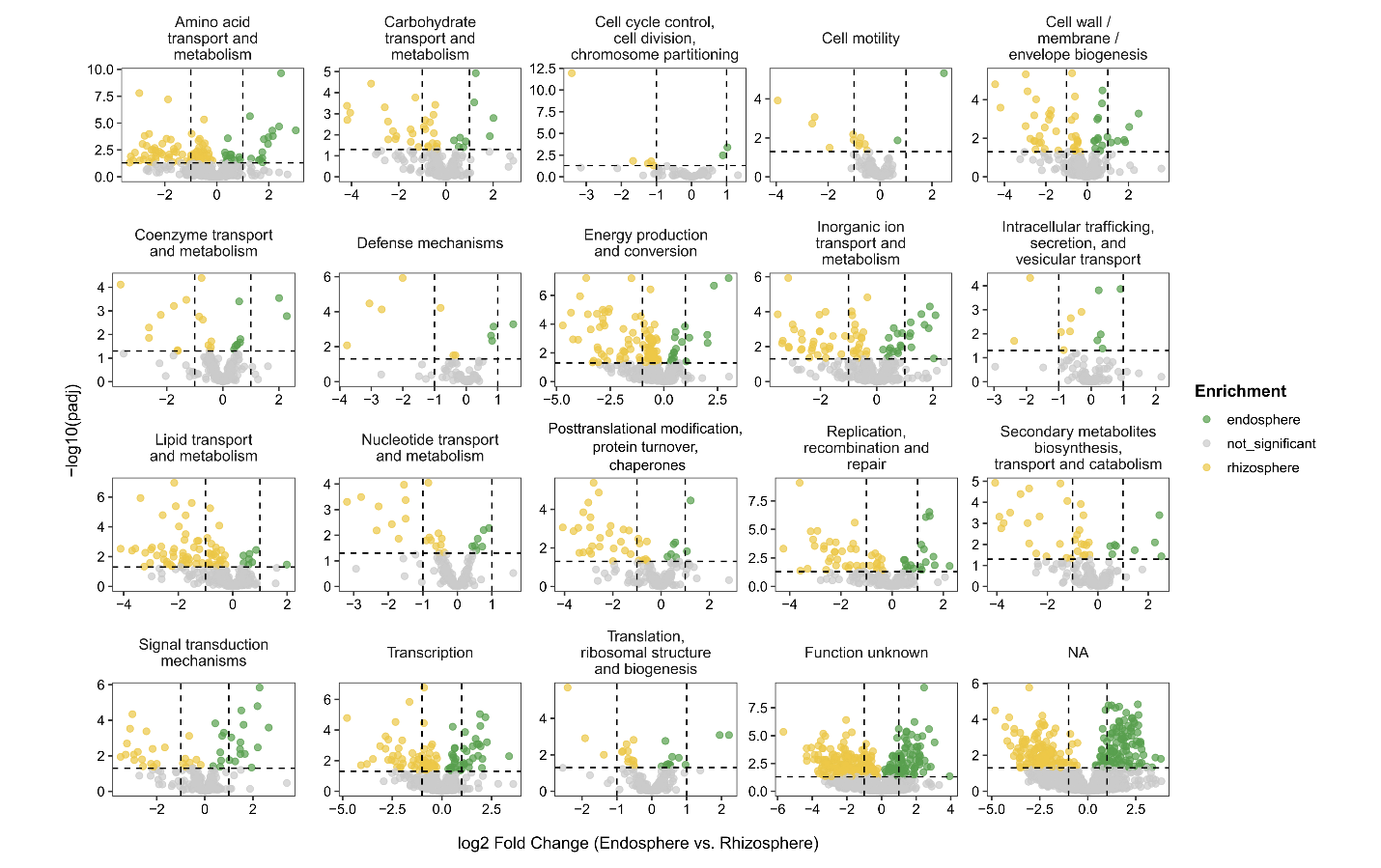

**Figure S5. (A)** Differential representation of orthogroups across COG functional categories in endosphere and rhizosphere compartments. Volcano plots show log₂ fold change (endosphere vs. rhizosphere) versus adjusted p-value from limma-voom analyses of raw orthogroup counts. Orthogroups enriched in the endosphere (log₂FC > 0, FDR < 0.05) are shown in green, those enriched in the rhizosphere (log₂FC < 0, FDR < 0.05) in yellow, and non-significant orthogroups in gray. Vertical dashed lines indicate log₂FC thresholds of ±1 and the horizontal line the FDR threshold of 0.05.
